# Microleakage in 5Y-TZP zirconia ceramic crowns and the influence of deep margin elevation: a laboratory investigation

**DOI:** 10.1007/s00784-026-06950-4

**Published:** 2026-06-03

**Authors:** Lucas Jansen, Matthias Kern, Sebastian Wille, Philipp Schadte, Nicole Passia

**Affiliations:** 1 Doctoral student, Department of Prosthodontics, Christian-Albrechts University of Kiel, University Hospital Schleswig-Holstein, Campus Kiel, Arnold-Heller-Strasse 3, House B, 24105 Kiel, Germany; 2 Department of Prosthodontics, Propaedeutics and Dental Materials, School of Dentistry, Christian-Albrechts University, Arnold-Heller-Strasse 3, House B, 24105 Kiel, Germany; 3 Department of Prosthodontics, Christian Albrecht University of Kiel, University Hospital Schleswig-Holstein, Campus Kiel, Arnold-Heller-Strasse 3, House B, 24105 Kiel, Germany; 4 Department of Material Science, Faculty of Engineering, Kiel University, Kaiserstr. 2, 24143 Kiel, Germany; 5 Department of Prosthodontics, Faculty of Medicine Carl Gustav Carus, Technische Universität Dresden, Fetscherstr. 74, 01307 Dresden, Germany

**Keywords:** Proximal box elevation, Deep margin elevation, Crown, Foundation restoration material

## Abstract

**Objectives:**

To evaluate the effect of deep margin elevation (DME) on microleakage of 5Y-TZP ceramic crowns after thermomechanical loading.

**Materials and methods:**

Forty extracted molars were allocated to five groups (*n* = 8). Standardized mesio-occluso-distal (MOD) tooth preprations were performed in all teeth and restored using two adhesive systems (one-bottle or two-bottle) in combination with two foundation restoration materials (auto-polymerizing or dual-polymerizing). Thirty-two molars received DME, while eight served as a control without DME. Following standardized tooth preparation, 5Y-TZP zirconia crowns were fabricated and bonded using an auto-polymerizing luting resin. While the specimens were stored in water for 150 days, they were subjected to thermomechanical loading with 37,500 thermal cycles (5 °C/55°C) and 1,200,000 mechanical cycles at 98 N in a chewing simulator. After loading, the specimens were immersed in fuchsine dye for 48 h, sectioned sagittally, and evaluated for dye penetration under an optical microscope.

**Results:**

Significant differences in microleakage were observed at the foundation restoration margins. The dual-polymerizing foundation restoration material demonstrated significantly lower microleakage than the auto-polymerizing material. The one-bottle adhesive system performed significantly better than the two-bottle system. No microleakage was detected at the crown margin in any experimental group.

**Conclusion:**

Within the limitations of this laboratory study, the use of a dual-polymerizing foundation restoration material in combination with a one-bottle adhesive system showed favorable results for DME.

**Clinical relevance:**

Compared with surgical crown lengthening or orthodontic extrusion, DME may reduce patient morbidity and treatment time. Clearfil DC Core, in particular, demonstrated low microleakage at DME margins, with the choice of adhesive system significantly influencing outcomes.

## Introduction

When teeth are structurally compromised, for example due to extensive caries or fractures, single crowns may be required to reestablish function and structural integrity. In this case, the biological width, as well as technical recommendations with regard to the restorative material have to be considered [1].

The biological width refers to the vertical dimension between a restoration margin and the alveolar bone, comprising the junctional epithelium and the supracrestal connective tissue attachment. It should not be violated during tooth preparation, as this may result in gingival recession or inflammation [1, 2]. However, subgingival defects frequently occur, particularly in premolars and molars, often as a result of proximal caries. In addition to considerations of biological width, restoring subgingival defects remains challenging, especially with regard to adhesive procedures, impression taking or rubber dam isolation.

Therefore, in some cases, surgical crown lengthening or orthodontic extrusion of the abutment tooth may be required as part of preprosthetic treatment [3–5]. As both procedures can have disadvantages such as pain, hypersensitivity, root resorption, and prolonged treatment time [2, 3, 6], alternative treatment options should be considered.

Several studies have therefore investigated to which extent deep subgingival defects can be restored using adhesive procedures without the need for surgical crown lengthening or orthodontic extrusion [7–10].

Deep margin elevation (DME) is a procedure in which an initially subgingival restoration margin is elevated to a supragingival level using composite resin.

In addition, DME can be used to relocate deep preparation margins to an easily accessible level, thereby enabling precise digital impressions and working within a CAD/CAM workflow [11].

Various laboratory studies have investigated the marginal quality of indirect ceramic restorations with deep margin elevation (DME) using different adhesive systems [8–10, 12, 13]. In some investigations, no significant differences in the marginal quality of indirect restorations were found compared to direct bonding to dentin, when DME was performed adequately. However, other studies have reported lower microleakage scores for restorations without DME than for those with DME [14, 15]. According to the current literature, three-step adhesive systems appear to be favorable for deep margin elevation [12, 16, 17].

Clinical investigations on the use of DME in combination with full-coverage crowns are scarce. In a recent clinical study by Aziz et al., the 10-year survival rates of teeth restored with lithium disilicate ceramic crowns following DME were comparable to those of a control group without DME [18].

Another clinical study compared DME with surgical crown lengthening [19]. After 12 months, both procedures were considered clinically successful, with favorable biologic responses. However, Mugri et al. concluded, that there is a lack of high-quality trials examining the differences between surgical crown lengthening and deep margin elevation with long-term follow-up [20].

Based on current knowledge, the effects of DME on the marginal quality of indirect restorations with localized subgingival defects are contradicting [21].

While some studies reported no reduction in marginal integrity [13, 22], others found reduced marginal integrity following DME [14, 15]. These contradicting results were confirmed by a systematic literature review on DME [21].

To date, the influence of DME using different foundation restoration materials and adhesive systems on the microleakage of zirconia ceramic crowns has not been investigated under laboratory conditions.

## Aim of study

The aim of the present study was to evaluate the microleakage of zirconia ceramic crowns (5Y-TZP) using the deep margin elevation (DME) technique. Two different adhesive systems and two different foundation restoration materials were investigated.

The null hypothesis of this study was, that there would be no differences between the adhesive systems (one-bottle or two-bottle) or the foundation restoration materials (auto-polymerizing or dual-polymerizing) with respect to the marginal quality of the DME and the crowns after thermomechanical loading.

## Materials and methods

### Tooth preparation, fabrication and cementation of crowns

For the present investigation, 40 extracted, caries-free human third molars were used. All specimens were prepared by a single operator to ensure consistent methodology. After extraction, the teeth were stored in a thymol solution for 5–7 days. Subsequently, all teeth were carefully cleaned of residual soft tissue and stored in distilled water in an incubator at body temperature until further use.

The teeth were coated with an artificial periodontal membrane made of of gum resin (Anti-Rutsch-Lack, Wenko-Wenselaar, Hilden, Germany) with a thickness of 0.25 mm to simulate the periodontium, following a protocol based on previous studies [23]. Subsequently, all specimens were embedded with their longitudinal axis oriented vertically in a custom-made mold using an auto-polymerizing polyester resin (Technovit 4000, Kulzer GmbH, Germany), following a standardized protocol described in previous studies [24, 25]. The gingival course was modeled 2 mm below the cementoenamel junction.

Standardized mesio-occluso-distal (MOD) preparations with a depth of 2 mm and a width of 3 mm were perforemd. Thirty-two randomly selected molars received proximal preparations extended 1 mm below the cementoenamel junction, later representing the DME, with dimensions of 1.5 mm in mesiodistal and 3 mm in bucco-lingual direction.

**Fig. 1:**
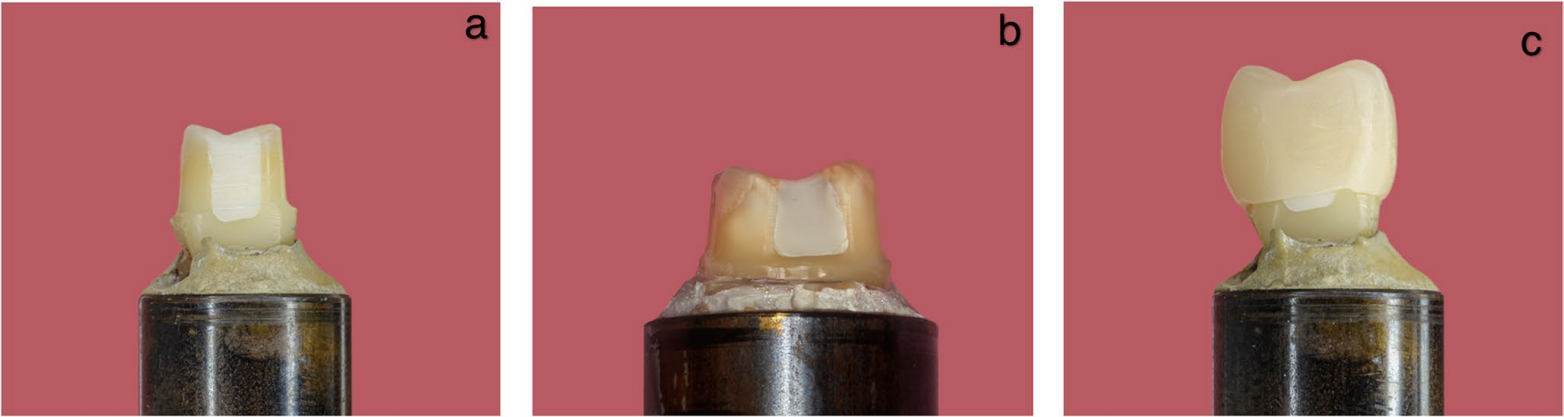
**a** Specimen experimental group 1 with DME; Fig. 1b Control group without DME; Fig 1c Specimen experimental group1 with DME after crown cementation

These 32 molars were randomly assigned to four experimental groups (*n* = 8), following a previously described study design, in which a sample size of eight specimens per group was considered sufficient to detect statistically significant differences [26, 27]. For the foundation restoration of the four experimental groups, two different adhesive systems and two different foundation restoration materials were used in the following combinations:

Group 1: Two-bottle Adhesive system: Clearfil SE Bond (Clearfil SE Bond, Kuraray), auto-polymerizing foundation restoration material: Clearfil Core (Clearfil Core, Kuraray), (Fig. 1a)

Group 2: One-bottle adhesive system: Universal Bond Quick (Unersal bond Quick, Kuraray), auto-polymerizing foundation restoration material: Cleafil Core.

Group 3: Two-bottle adhesive system: Clearfil SE Bond, dual-polymerizing foundation restoration material: Clearfil DC Core (Clearfil DC Core, Kuraray).

Group 4: One-bottle adhesive system: Universal Bond Quick, dual-polymerizing foundation restoration material: Cleafil DC Core.

The remaining eight teeth also received MOD preparations, with margins located 1.5 mm above the cementoenamel junction. These specimens served as the control without DME and were assigned to group 5 (Fig. 1b). **c** Final specimen of the experimental group 1 with DME.

Further details on the materials used are provided in Table 1. All materials were used in accordance with the manufacturers’ instructions.

**Table 1:** material used in the study

Material	LOT	Material composition according to the manufacturer
Clearfil Core New Bond (Kuraray, Hattersheim, Germany); auto-polymerizing foundation restoration material	000184	Catalyst paste: Bis-GMA, TEGDMA, silanated glass filler, colloidal silica, benzoyl peroxide Universal paste: Bis-GMA, TEGDMA, silanated silica filler, colloidal silica, accelerators
Clearfil SE Bond (Kuraray, Hattersheim, Germany); two-bottle adhesive system	000472	Primer: MDP, HEMA, hydrophilic aliphatic dimethacrylate, dl-camphorquinone, N,N-diethanol-p-toluidine, water Bond: MDP, Bis-GMA, HEMA, hydrophobic aliphatic dimethacrylate, dl-camphorquinone, accelerators, N,N-diethanol-p-toluidine, colloidal silica
Clearfil Universal Bond Quick (Kuraray, Hattersheim, Germany); one-bottle adhesive system	1D0389	MDP, Bis-GMA, HEMA, hydrophilic amide monomers, colloidal silica, silane coupling agent, sodium fluoride, dl-camphorquinone, ethanol, water, phenyl bis(2,4,6-trimethylbenzoyl)-phosphine oxide, accelerators
Clearfil DC Core Plus (Dentine) (Kuraray, Hattersheim, Germany); dual-polymerizing foundation restoration material	AR0523	Paste A: Bis-GMA, hydrophobic aliphatic dimethacrylate, hydrophilic aliphatic dimethacrylate, hydrophobic aromatic dimethacrylate, silanated barium glass filler, silanated colloidal silica, colloidal silica, dl-camphorquinone, benzoyl peroxide, initiators, pigments Paste B: triethylene glycol dimethacrylate, hydrophilic aliphatic dimethacrylate, hydrophobic aromatic dimethacrylate, silanated barium glass filler, silanated colloidal silica, aluminum oxide filler, accelerators
Panavia V5 Kit (A2 Universal) (Kuraray, Hattersheim, Germany); dual-polymerizing resin cement	000142	Paste: silanated barium glass filler, hydrophobic aromatic dimethacrylate, Bis-GMA, silanated fluoroaluminosilicate glass filler, hydrophilic aliphatic dimethacrylate, silanated titanium dioxide, TEGDMA, surface-treated aluminum oxide filler, colloidal silica, dl-camphorquinone, initiators, accelerators, pigments Tooth Primer: MDP, HEMA, hydrophilic aliphatic dimethacrylate, N,N′-dimethylaminoethyl methacrylate, accelerators, water Ceramic Primer Plus: 3-methacryloxypropyl trimethoxysilane, MDP, ethanol

When using the two-bottle adhesive system, the enamel areas were selectively etched with 35% phosphoric acid for 30 s (s) (K-ETCHANT syringe, Kuraray, Hattersheim, Germany). The self-etching primer was applied to the tooth surface with a microbrush (Young Innovations Europe GmbH, Germany) for 20 s and air-dried using oil-free air. The adhesive was applied to the entire Class II MOD preparation, gently air dried to create a uniform film, and light-cured for 10 s using a polymerization light with a wavelength of 440–480 nm and an intensity of 1200 mW/cm² (RADII-CAL; SDI).

When using the one-bottle adhesive system, the enamel areas were likewise selectively etched with 37% phosphoric acid for 30 s. The adhesive was applied to the entire Class II MOD preparation using a microbrush, gently air-dried for 5 s and light-cured for 10 s.

Afterwards, the respective foundation restoration material was applied. The auto-polymerizing foundation restoration material Clearfil Core was mixed manually in a 1:1 ratio and inserted using the designated application syringe. After insertion, the material auto-polymerized for 7 min.

For the dual-polymerizing foundation restoration material Clearfil DC Core, the material was directly dispensed into the Class II MOD preparation in three increments using an auto-mixing tip. Each increment was light-cured for 20 s with subsequent auto-polymerization for 6 min. All teeth were prepared in a standardized manner using a paralleling device to ensure an adequate retention and resistance form, with a minimum axial wall height of 3 mm and a convergence angle of 3° to 6°. The preparation margins were located at the level of the cementoenamel junction, resulting in crown margins partly located in the foundation restoration material for the experimental groups (Fig. 1a). Diamond burs of medium grit (No. 878016, No. 837KR012; Komet, Gebr. Brasseler, Lemgo, Germany) were used at 40,000 rpm. To achieve a surface roughness of approximately 15 μm, the preparations were finished using fine-grit diamond burs at 20,000 rpm (No. 18878K014, No. 8837KR016; Komet, Gebr. Brasseler, Lemgo, Germany). Afterwards, specimens were stored in distilled water at body temperature. All prepared teeth were digitally scanned using a laboratory scanner (E4, 3Shape, Copenhagen, Denmark).

The crowns were digitally designed with a uniform thickness of 1 mm. Standardized plateaus (3 × 3 mm) were designed on the load-bearing cusps of all specimens at an angle of 30° to the tooth axis to later ensure standardized contact with the steatite spheres of the chewing simulator during masticatory loading. The crowns were milled from zirconia ceramic (5Y-TZP, Katana UTML, Kuraray Noritake) and sintered according to the manufacturer’s instructions.

Prior to cementation, the crowns were clinically evaluated for marginal and internal fit using dental loupes and an explorer. In cases of minor interference, fit was verified using Fit Checker (GC Europe N.V., Leuven, Belgium) and any interferances were adjusted.

The abutment teeth were cleaned with pumice. The intaglio crown surfaces were air-abraded with 50 μm alumina particles at 1.0 bar and subsequently cleaned in an ultrasonic bath with isopropanol for 3 min. The crowns were dried with oil-free air, Ceramic Primer Plus (Panavia V5, Kuraray Noritake) was applied and air-dried. Tooth Primer (Panavia V5, Kuraray Noritake) was applied to the abutment teeth for 20 s and air-dried.

The crowns were bonded using Panavia V5 (Kuraray Noritake) under a standardized load of 5 kg. After an initial light-curing for 5 s, excess luting resin was removed with a scaler, followed by additional light-curing for 10 s per surface. Residual excess material was removed after 3 min. using a scaler and polishing instruments (Fig. 1c).

Final polishing was performed using Sof-Lex discs (3 M Deutschland GmbH, Neuss, Germany), progressing from coarse to super-fine grit at a maximum speed of 10,000 rpm. Light pressure and continuous movement from the restoration margin towards the coronal direction were applied. Between each polishing step, the surfaces were rinsed using a multifunction syringe. Polishing and final inspection were performed under magnification using dental loupes.

### Fatigue test

The specimens were subjected to thermal cycling (5 °C/55°C) for 37,500 cycles over a period of 150 days and subsequently loaded in a chewing simulator (Willytec chewing simulator, Willytec) for 1,200,000 chewing cycles.

To simulate intraoral conditions, a loading frequency of 2.0 Hz with a lateral sliding movement of 0.3 mm toward the central fissure of the crowns was applied. The downward velocity was 30 mm/s, the upward velocity was 55 mm/s, and the vertical displacement was 6 mm. A load of 98 N was applied to simulate high physiological masticatory forces, following a protocol described in previous studies [26, 28, 29]. The antagonist was simulated using a steatite sphere with a diameter of 5 mm (CeramTec, Hoechst). The sphere was positioned to initially contact the standardized plateau before sliding 0.3 mm in a central direction, thereby avoiding contact with the opposing cusp. The contact point was verified using articulating foil prior to loading.

### Microleakage

After fatigue testing, the specimens were evaluated for microleakage.

For this purpose, the specimens were immersed in 2% basic fuchsin solution and stored at 36 °C for 48 h. Subsequently, they were rinsed, and superficial dye was carefully removed using rotary instruments and a toothbrush under running water. The roots were sectioned 2 mm below the crown margins, and all specimens were embedded in clear auto-polymerizing resin (Technovit 4071, Kulzer, Germany).

The specimens were sectioned using a precision saw (Primus; Walter Messner GmbH, Germany) to obtain three consecutive longitudinal slices in mesiodistal direction within the area of the foundation restoration, each with a thickness of 500–800 μm.

Dye penetration was evaluated at both, the foundation restoration interface and the crown interface using a light microscope (Wild M420, Wild Heerbrugg AG, Switzerland) at 16× magnification. The extent of microleakage was assessed using a standardized scoring system, as shown in Fig. 2a.

**Fig. 2a:**
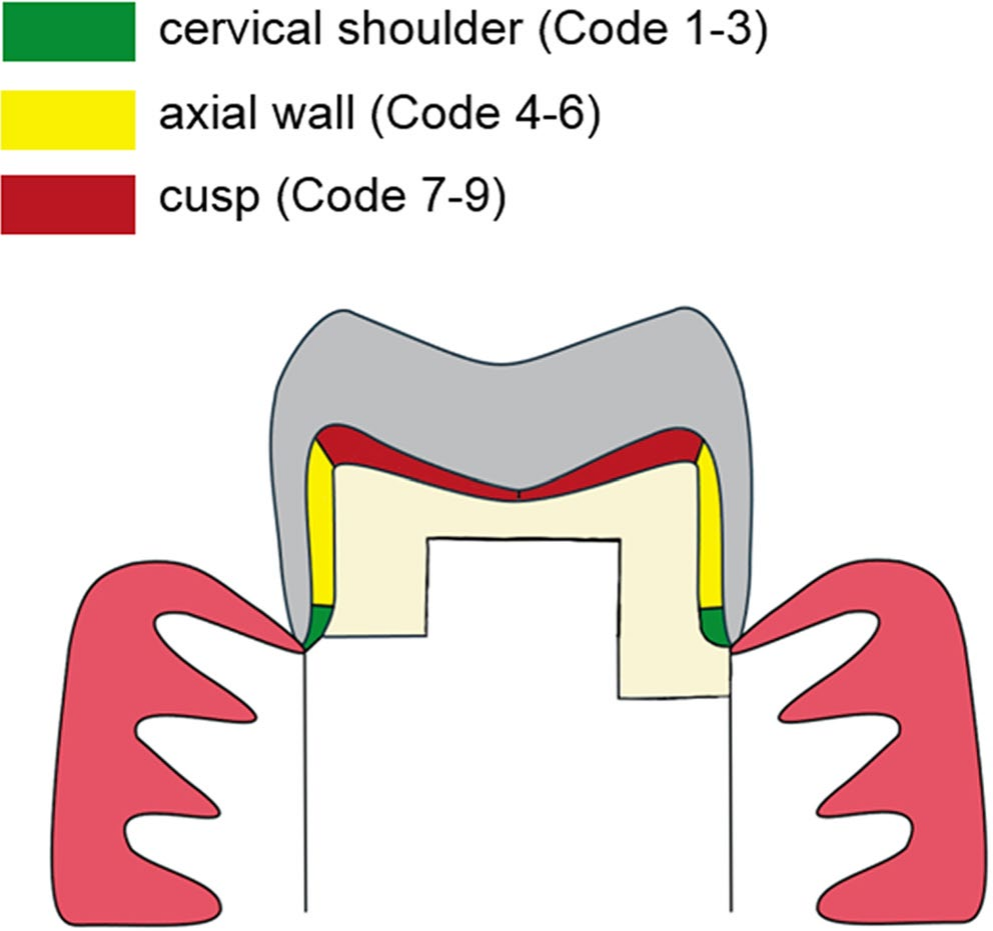
Schematic illustration of the microleakage score. Legend: 0 = no microleakage. 1 = microleakage up to 1/3 of the cervical shoulder. 2= microleakage up to 2/3 of the cervical shoulder. 3 = microleakage over the entire cervical shoulder. 4 = microleakage up to 1/3 of the axial wall. 5 = microleakage up to 2/3 of the axial wall. 6 = microleakage over the full length of the axial wall. 7 = microleakage up to the tip of the cusp. 8 = microleakage up to the middle of the cusp slope. 9 = microleakage up to the central fissure.

As part of preliminary experiments, two examiners were calibrated for the evaluation procedure. In the present study, the assessment of the specimens was subsequently performed by a single examiner. To verify the reliability of the assessments, randomly selected specimens were re-evaluated by the second calibrated examiner.

Dye penetration was assessed at two different interfaces (Fig. 2b), with the following penetration options:

**Fig. 2b:**
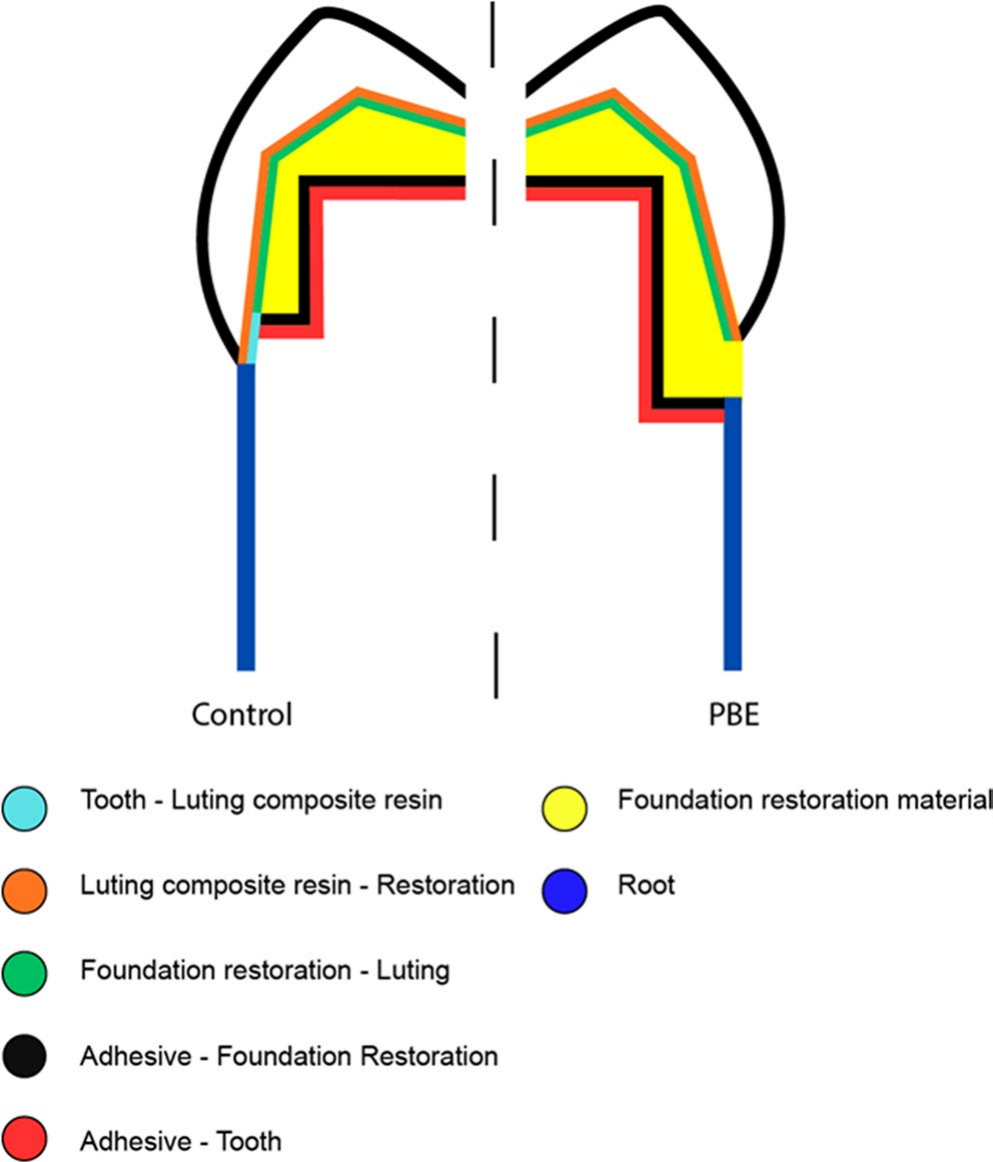
Schematic illustration of penetration possibilities

Adhesive and crown interface = crown margin:

tooth structure – luting composite (light blue).

luting composite – crown (orange).

foundation restoration – luting composite (green).

Adhesive and tooth interface = foundation restoration margin:

adhesive – foundation retoration (black).

adhesive – tooth (red).

Two interfaces were evaluated for microleakage: the crown margin (located either within the foundation restoration material in the experimental groups or within sound dentin in the control group) and of the foundation restoration margin (DME). By analyzing these two interfaces, clinically relevant microleakage could be revealed.

Statistical analysis was performed using SPSS software (version 29; SPSS Inc., Chicago, IL, USA). As data were not normally distributed according to the Shapiro–Wilk test, nonparametric tests were subsequently used. The Kruskal–Wallis test was used to assess differences between groups. For pairwise comparisons, the Mann–Whitney U test with Holm–Bonferroni correction was used. The level of statistical significance was set at α = 0.05.

Randomly selected replicas from all groups (before and after loading) with mean microleakage scores were sputter-coated with a 10 nm gold layer and examined using scanning electron microscopy (SEM; Zeiss Supra 55 VP, Carl Zeiss, Germany) at an accelerating voltage of 10 kV to evaluate differences before and after thermomechanical loading.

## Results

**Fig. 3:**
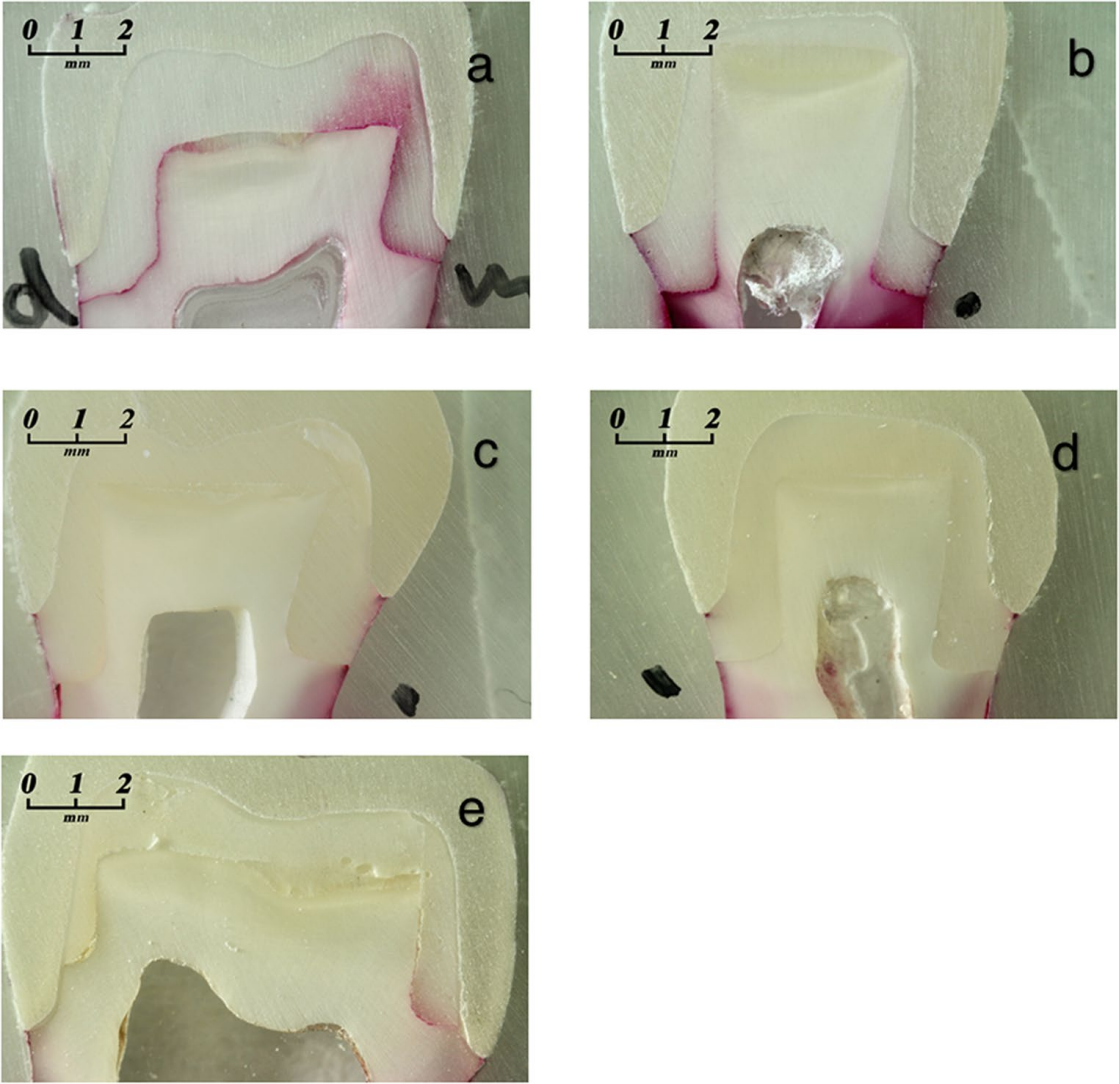
**a**-**e** Typical section of a specimen from each group (basic fuchsin stain; original magnification x 16) **a** group 1 with an auto-polymerizing foundation restoration material and the two-bottle adhesive system **b** group 2 with an auto-polymerizing foundation restoration material and the one-bottle system **c** group 3 with a dual-polymerizing foundation restoration material and the two-bottle system **d** group 4 with a dual-polymerizing foundation restoration material and a one-bottle system **e** group 5 with no DME (control group)

Statistically significant differences in microleakage were observed between the two bonding interfaces, as shown in Table 2. The crown margins exhibited significantly lower microleakage scores compared to the foundation restoration margins (*p* ≤ 0.05). At the crown margin, group 4 showed significantly higher microleakage scores than the other experimental groups (*p* ≤ 0.05). At the foundation restoration margin, significant differences were observed between the groups with auto-polymerizing foundation restoration material (groups 1 and 2; Fig. 3a, b) and those with dual-polymerizing foundation restoration material (*p* ≤ 0.05).

**Table 2:** Evaluation of the color penetration along the adhesive interfaces according to the microleakage score scale. Median, means and standard deviations (s.d.) are given in numbers analogous to the penetration code. Statistically different medians (*p* ≤ 0.05) are indicated by different superscript uppercase letters (witin a column), or by subscript lowercase letters (within a row)

Group	Crown interface			Foundation restoration interface		
	Median	Mean	s.d.	Median	Mean	s.d.
1. Auto-polymerizing foundation restoration material + two-bottle system	0.00 ^B^_b_	0.56	0.77	7.50 ^A^_a_	6.52	2.41
2. Auto-polymerizing foundation restoration material + one-bottle system	1.00 ^AB^_b_	0.83	0.98	4.00 ^B^_a_	4.33	1.28
3. Dual-polymerizing foundation restoration material + two-bottle system	0.00 ^B^_b_	0.54	0.77	1.00 ^CD^_a_	1.81	1.88
4. Dual-polymerizing foundation restoration material + one-bottle system	1.00 ^A^_a_	1.08	0.80	0.50 ^D^_a_	1.23	1.45
5. Control, auto-polymerizing foundation restoration + two-bottle system, No DME	0.00 ^C^_b_	0.06	0.25	3.00 ^C^_a_	2.90	2.90

Groups 1, 2, and 4 (Fig. 3d) differed significantly from the control group (group 5, Fig. 3e). Higher microleakage scores were observed in groups 1 and 2, whereas group 4 exhibited significantly lower microleakage scores (*p* ≤ 0.05).

When comparing the two adhesive systems, the one-bottle system exhibited lower microleakage scores than the two-bottle system when used with the same foundation restoration material at the DME margin (*p* ≤ 0.05).

**Fig. 4:**
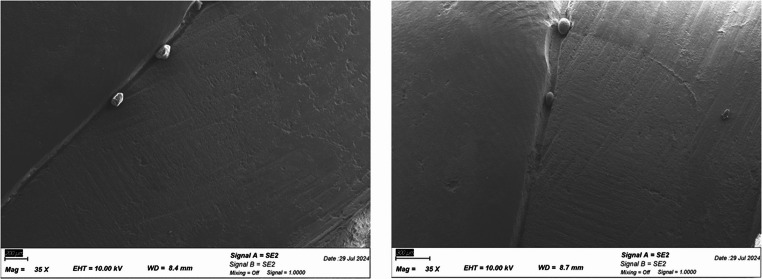
**a** SEM analysis of a specimen with DME before loading (left); Fig. 4b SEM analysis of the same specimen after loading (right)

The SEM analysis of the specimens before and after loading added no additional information to the findings obtained from the dye penetration test (Fig. 4a, b).

## Discussion

The first null hypothesis, that there would be no differences between the adhesive systems with regard to the quality of DME, was rejected, as the groups using the one-bottle adhesive system revealed lower microleakage scores compared to those using the two-bottle adhesive system. This finding may be attributed to the lower technique sensitivity of one-bottle adhesive systems. A previous investigation comparing Universal Bond Quick with two-bottle adhesive systems reported, that Universal Bond Quick achieved comparable or higher shear bond strength compared to the two-bottle controls [30].

Other studies found a three-step adhesive system to be superior [12, 16].

The second null hypothesis, that there would be no differences between the foundation restoration materials with respect to the quality of DME, was rejected. Notably, the groups using the auto-polymerizing foundation restoration material exhibited higher microleakage scores than those using the dual-polymerizing material.

This could be due to the material processing. The auto-polymerizing material was mixed manually, whereas the dual-polymerizing material was applied using automixing syringes, potentially resulting in better material properties.

However, a previous study whith dual-polymerizing foundation restoration materials polymerizing either chemically alone or in combination with light-curing reported, that dual-polymerization resulted in significantly higher mean bond strength [31]. Another investigation compared the median tensile bond strength of a dual-polymerizing resin material and an auto-polymerizing resin material to dentin and similarly demonstrated that the dual-polymerizing material provided more durable bond strength [32]. These studies suggest, in accordance with the results of the present investigation, that dual-polymerizing foundation restoration materials may exhibit higher bond strength to dentin than auto-polymerizing materials [31–33].

Crown margin discrepancies or bonding failures may allow microorganisms to penetrate the adhesive interfaces. These microorganisms can lead to secondary caries or pulpal inflammation [34].

The results of the present study indicate, that the adhesive bond of the crown appears to be stable on DME, as no microleakage was observed at the crown margin. In this context, slightly increased microleakage scores are most likely attributed to reduced crown fit rather than true microleakage. This finding is consistent with previous investigations showing that bonding ceramic to composite yields excellent results [29]. The special structure of dentin makes bonding difficult [35]. Bonding to enamel provides higher and more predictable adhesion than bonding to dentin due to enamel’s high mineral content and homogeneous structure, which allow for optimal micromechanical interlocking. In contrast, dentin’s higher organic content, greater tubule density, and intrinsic moisture make adhesive penetration and hybrid layer formation more challenging [36]. Therefore, restoration margins located in dentin, such as the foundation restoration margins in the experimental groups, are more susceptible to microleakage and, consequently, to sensitivity, discoloration at the adhesive interface and secondary caries [37–39]. The adhesive bond between the foundation restoration material and dentin was weaker than the adhesive bond of the crowns in DME, resulting in greater detectable microleakage at the DME interface, which is consistent with previous literature [7, 23].

Nevertheless, the selected aging protocol is also relevant for the interpretation of the experimental results. When simulating the aging of crowns, 1,200,000 chewing cycles and 37,500 thermocycles correspond to approximately 4–5 years of clinical service [40]. Restorations are typically subjected to thermomechanical loading in a chewing simulator, including thermocycling between 5 °C and 55 °C. However, both, the number of mechanical loading cycles and the number of thermal cycles vary considerably in the literature [8–10].

Compared to other investigations, the present study showed increased microleakage at the crown margins in groups 1 and 2 [8, 9, 14, 41]. This finding may be influenced by the extended aging protocol, which included 37,500 thermocycles and 1,200,000 chewing cycles following 150 days of water storage compared to other investigations [9, 10, 42]. In addition, the higher load of 98 N applied in the present study, compared to the more commonly used 49 N, may have contributed to these findings [8–10, 22].

Different results may also be attributed to the method of analysis, such as dye penetration assessment or evaluation using SEM [41, 42]. Dye penetration followed by light microscopic evaluation is a standard method for detecting marginal discrepencies in laboratory investigations, whereas SEM evaluation alone appears to have limited sensitivity [43]. This is consistent with the findings of the present study, as SEM analysis of additional replicas of the DME and crown margins before and after aging did not reveal any additional information beyond that obtained from dye penetration analysis.

Interestingly, in some cases, microleakage at the foundation restoration interface was also observed in the control group, in which the crown margins were located in sound dentin (Fig. 3e). Although dye penetration at the crown margin was not clearly visible in the examined section, the dye likely penetrated the crown margin adjacent to the section plane and subsequently migrated beneath the foundation restoration material.

As both, the foundation restorations and the crowns in the control group were bonded to dentin, this finding further highlights the vulnerability of bonding to dentin. It should be considered that, in the experimental groups, freshly prepared dentin was immediately sealed with the foundation restoration material. Although a similar procedure was applied in the control group, the foundation restoration margin was completely covered by the crown. The crown margin was evaluated later. Consequently, the dentin at the crown margin was prepared in advance but restored later after crown fabrication.

Several authors have reported that immediate dentin sealing enhances the bond strength of indirect restorations, as freshly prepared dentin provides a more favorable substrate for subsequent bonding [44, 45].

However, bonding to dentin is generally considered more challenging and less predictable than bonding to enamel [35, 46] or composite [29]. This, once again, underscores the importance of a meticulous approach to adhesive bonding. Due to the pattern of the dentinal tubules, dye penetration limited to the cervical shoulder of the preparation is considered less critical than penetration extending to the axial wall [47].

This should be taken into account when assessing the clinical relevance of microleakage.

As demonstrated in the present laboratory study, the choice of materials used for DME appears to influence the marginal quality of restorations, which is consistent with findings from previous studies [48]. The results of the present study indicate that clinical complications may be expected in groups using auto-polymerizing foundation restoration materials.

A strength of this laboratory study is the extensive aging and loading protocol, which corresponds approximately to a clinical service of 4–5 years.

A limitation of the present study is that the specimens were assigned exclusively to either the experimental or the control group. Alternatively, a split-tooth design could have been applied, in which the experimental and control conditions were evaluated within the same specimen. Deep margin elevation could have been applied on the mesial side and the control condition (no DME) on the distal side, or vice versa. In a longitudinal section, both conditions could thus have been assessed within a single specimen.

## Conclusion

Within the limitations of the present laboratory study, dual-polymerizing foundation restoration materials demonstrated favorable microleakage outcomes when used for deep margin elevation (DME). DME appears to be a promising approach for the restoration of deep proximal defects.

Furthermore, the one-bottle adhesive system performed slightly better than the two-bottle adhesive system. However, well-designed clinical studies with long-term follow-up are needed to confirm these findings.

## Data Availability

We newly generated data from laboratory experiments.

## Declarations

**Ethics declaration**: The institutional review board of the study center approved the use of extracted teeth in this laboratory investigation (ethics committee of the Christian-Albrechts-University at Kiel, AZ: D528/19).
